# Open reduction and internal fixation versus percutaneous transverse Kirschner wire fixation for single, closed second to fifth metacarpal shaft fractures: a systematic review

**DOI:** 10.1007/s00068-015-0507-y

**Published:** 2015-03-14

**Authors:** A. P. A. Greeven, S. Bezstarosti, P. Krijnen, I. B. Schipper

**Affiliations:** 1 Department of Trauma Surgery, Leiden University Medical Centre, Leiden, The Netherlands; 2 Department of Surgery, HagaHospital, The Hague, The Netherlands

**Keywords:** Closed metacarpal fracture, Single shaft fracture, Operative treatment, Open reduction and internal fixation, Percutaneous fixation, Kirschner wire

## Abstract

**Purpose:**

Open reduction and internal fixation (ORIF) of single, closed metacarpal shaft fractures is increasingly preferred over closed reduction and percutaneous fixation (K-wire). The aim of this systematic review is to determine whether the preference for ORIF can be substantiated based on the available literature regarding the functional outcome and complications after surgery.

**Methods:**

A systematic review was performed using a computer-based search on MedLine and Embase, following the preferred reporting items for systematic and meta-analyses guidelines.

**Results:**

Five non-comparative studies were found. Two studies reported on 36 ORIF-treated patients. Three studies reported on 65 K-wire-treated patients. Complications were reported in 8 ORIF-treated patients (22 %) and in 23 K-wire-treated patients (35 %). Functional outcome was generally reported as good for both techniques. Nonetheless functional impairment requiring reoperation was reported in 6 ORIF-treated patients (17 %) and in none of the K-wire-treated patients.

**Conclusions:**

Although for both techniques good functional outcomes were reported, the significance of the functional impairment after ORIF requiring reoperation suggests ORIF to be a less favorable technique for single, closed metacarpal shaft fractures.

## Introduction

Metacarpal fractures are among the most common fractures of the skeletal system and account for 36 % of hand and wrist fractures [2, 9, 14, 15]. The peak incidence of metacarpal shaft fractures is between 20 and 40 years and results in significant societal costs [11]. The majority of metacarpal shaft fractures can be treated conservatively [14]. Numerous indications for operative treatment include malrotation, angulation, longitudinally shortening, multiple fractures and fractures with associated soft tissue injuries or bone loss [2, 3, 7, 8, 14, 18, 20]. With the introduction of new fixation techniques for metacarpal fractures in the last 25 years, open reduction and internal fixation (ORIF) gained increasing popularity, because stable ORIF fixation allows early mobilization [6, 19]. The reasons for surgeons to decide for open reduction and internal fixation also included the improvement of materials and instruments, better understanding of biomechanical principles of internal fixation, and the availability of antibiotics to reduce infection. A well-known alternative surgical treatment options is closed reduction and percutaneous fixation with Kirschner wires (K-wires) [3, 14].

This systematic review was performed to determine the functional outcome and postoperative complication for both these surgical techniques in the treatment of single, closed metacarpal shaft fractures. This review aims to determine whether the preference for ORIF can be substantiated based on available data in the literature in terms of functional outcome and complications.

## Methods

A systematic review was performed following the Preferred Reporting Items for Systematic Reviews and Meta-Analyses guidelines, including (1) a systematic search of the literature, (2) selection of studies, (3) recording of study characteristics, (4) assessment of methodological quality of studies, and (5) extraction and comparison of clinical outcomes [10].

### Search strategy

The literature search was conducted in both MedLine and Embase on September 12th 2014. The search strategies were developed by a trained medical librarian and included combinations of different terms and synonyms for extra-articular metacarpal fractures and surgical treatment. The detailed search strategies are described in the “Appendix”.

### Selection of studies

After removal of duplicate studies from the MedLine and Embase literature searches, the title and abstract of the remaining studies were screened to evaluate if they met the following criteria: (1) Language: English or German. (2) Study design: comparative (randomized or non-randomized), prospective or retrospective studies. (3) Population: Humans with a single shaft fracture located in the second, third, fourth or fifth metacarpal. (4) Intervention: ORIF and/or percutaneous transverse K-wires. (5) Outcome: hand function, consolidation and/or complications.

**Fig. 1:**
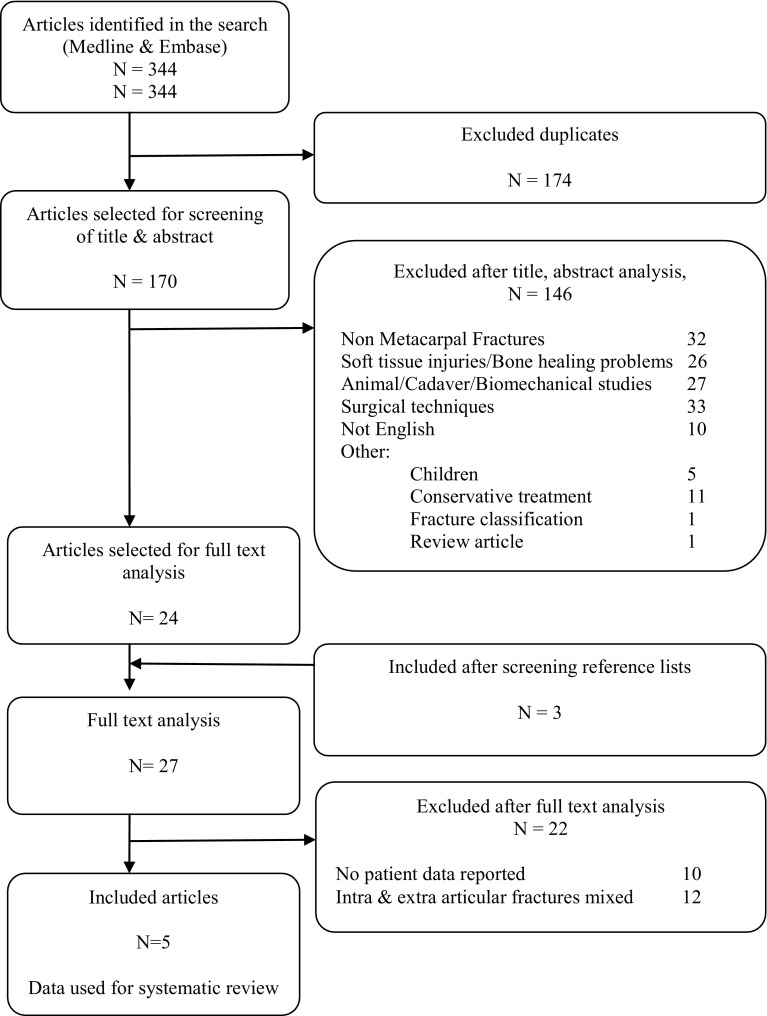
Flowchart of selected articles

Of the selected abstracts, the full-text articles were screened using the same inclusion criteria. The reference lists of selected articles were screened for additional relevant studies (Fig. 1).

### Recording of study characteristics

The following study characteristics were extracted from the five selected full-text articles: author, title, publication year, country of origin, study design, number of participants, type of surgical treatment, complications and follow-up period (Tables 1, 2).

**Table 1:** Characteristics of included studies

	Study	Year	Country	Study design	Fixation	No. of patients	Follow-up
1	Ozer	2008	USA	Prospective cohort	ORIF	14	19 (12–219)
2	Westbrook	2008	United Kingdom	Retrospective cohort	ORIF	22	180 (100–240)
3	Galanakis	2003	Greece	Retrospective cohort	K-wire	11	12 (120
4	Paul	1994	United Kingdom	Prospective cohort	K-wire	22	*
5	Sletten	2012	Norway	Retrospective cohort	K-wire	32	128 (68–156)

* Not specified, follow-up reported until full consolidation

**Table 2:** Data extracted from included articles

Study	1 (*n* = 14)	2 (*n* = 22)	3 (*n* = 11)	4 (*n* = 22)	5 (*n* = 32)
Mean age (years)	28 (19–47)	25 (14–79)	43 (18–64)	*	30 (19–50)
Pre-operative angulation	14 (0–82)	29 (16–62)	37 (32–42)	36 (32–40)	35 (1–69)
Fracture location					
MC II	0	0	*	4	0
MC III	0	0		2	0
MC IV	3	0		8	11
MC V	11	22		8	21
Fixation	Plate-screw	Plate-screw	Transverse K-wire (size: 1.4 mm)	Transverse K-wire (size: 1.4 mm)	Transverse K-wire (size: *)
Immobilization (days)	Bulky dressing 13 (12–14)	Non applied 0	Cast 7 (7)	Non applied 0	Cast 35 (28–49)
Follow-up (weeks)	19 (12–219)	180 (100–240)	12 (12)	*	128 (68–156)
Postoperative angulation	0	*	0	2.2 (0–10)	10 (2–19)
Postoperative shortening (mm)	0	*	0	*	0
TAM/function	225 (150–270)	*	Full function	Full function	264 (250–296)
DASH	8.07 (1–28)	5 (1–44)	*	*	1 (0–39)^b^
Complications					
Infection	0	2	0	8	8
Impairment	2	3	0	0	0
Pain	0	1	0	0	0
Other	0	0	0	0	3^c^

* Not specified

^a^as measured on lateral X-ray

^b^Patients with higher DASH scores had suffered other injuries in their upper limbs during follow-up period

^c^Fracture at former K-wire site in uninjured metacarpal

### Assessment of methodological quality

The risk of bias was assessed following the instructions by Spindler et al. [17] within and between studies and the level of evidence of the selected studies was assessed.

Data-extraction and comparison of clinical outcomes were reviewed. The following data was extracted from the selected full-text articles: functional outcome, complications (reoperation, infection, delayed/non-union and failure of fixation) and postoperative cast immobilization. Delayed union and non-union were defined as lack of bony consolidation on radiographs at 3 and 6 months, respectively [1, 5].

Two researchers performed steps 2–5 independently. During step 2, disagreement about selection of studies was resolved by study inclusion. Disagreement during steps 3–5 was resolved by discussion.

## Results

### Study selection

The search identified 158 articles in Medline and 186 articles in Embase. After removing 174 duplicate studies, the title and abstract of the remaining 170 articles were screened. A total of 24 articles were selected for full-text reading. By screening of the references of these 24 articles another three potentially relevant articles were found. After full-text examination of these 27 articles, 22 articles were excluded as these articles did not provide patient data or did not meet the selection criteria. The remaining five articles were included in the systematic review and the reported data in these articles were used for analyses (Fig. 1).

### Study characteristics

No randomized or non-randomized studies comparing ORIF with K-wire fixation were found. The selected articles described three retrospective and two prospective patient cohorts, including two that had been treated with ORIF [12, 19] and three with K-wires [6, 13, 16] (Table 1). One article reported on patients treated with intra-articular as well as on patients treated with metacarpal shaft fractures [19]. From this study the separate results of the metacarpal shaft fractures could be extracted and were used for this review. In total, the five articles reported on outcomes of 36 metacarpal shaft fractures treated with ORIF in 36 patients and on 65 metacarpal shaft fractures treated with transverse K-wire fixation, in 65 patients (Table 1).

All studies included patients with single, closed unstable metacarpal shaft fractures.

### Functional outcome

Functional outcome was reported in all five studies and measured by total active motion (TAM, normal range 290–310) or by a disability arm shoulder (DASH) score (Table 2). The functional outcome of patients treated with ORIF was reported to be generally good, with a TAM between 150° and 270° or a DASH score between 1 and 44 (Table 2). All K-wire-treated patients were reported to have good functional outcome, except one. This patient was reported to have an extension loss of 10°. The other 64 patients were reported to have full function or to have a range of motion (ROM) and grip strength equalling that of the contra-lateral, uninjured hand (Table 2).

### Complications

In the ORIF-treated patients a total of 8 patients (22 %) were reported to have had a complication after operative treatment (details provided in Table 3). Six of these patients (17 %) experienced major functional impairment from these complications and required a reoperation. In the K-wire-treated patients a total of 23 (35 %) were reported to have encountered a complication (Table 3). None of these complications resulted in functional impairment or required reoperation.

**Table 3:** Details on complications and Reoperations per treatment

Complication	ORIF (36 fractures)		K-wire (65 fractures)	
	No. with complication	No. of reoperations	No. with complication	No. of reoperations
Delayed union	0	0	0	0
Non-union	0	0	0	0
Fixation failure	0	0	0	0
Stiffness/tenolysis	5 (14 %)	5 (14 %)	0	0
CRPS	0	0	0	0
Infection	2 (6 %)	0	16 (25 %)	0
Pain	1 (3 %)	1 (3 %)	0	0
Skin irritation	Not reported	Not reported	4	0
Cosmetic deformity	Not reported	Not reported	0	0
New fracture	0	0	3 (5 %)^a^	0
Total	8 (22 %)	6 (17 %)	23 (35 %)	0

0 = articles report no such complication occurred

*Not reported* no mention was made in the reviewed article about the type of complication or reoperation mentioned

^a^Fracture after new trauma during follow-up, at former K-wire location in non-fractured metacarpal

### Infections

In the ORIF group infections occurred in 2 patients (6 %). Both patients were treated with oral antibiotics (Table 3). In the K-wire-treated patients superficial skin infection was reported in 16 patients (25 %). Nine of those were treated with oral antibiotics (14 %) and 7 with removal of K-wires (11 %) (Table 3).

### Non-union/delayed union

Non-union or delayed union was not reported in any of the five studies (Table 3).

### Failure of fixation

In none of the ORIF and K-wire-treated patients did a failure of fixation occur (Table 2).

### Stiffness/tenolysis

In the ORIF group impairment of function as a result of stiffness was reported for 5 patients (14 %) (Table 2). Causes for stiffness were not specified. In all 5 patients (14 %) this impairment was reported to require a reoperation because of persistent functional deficit. No functional impairment was reported for the K-wire-treated patients.

### Other findings

One ORIF study reported on bulky dressing for 12–14 days postoperatively [12]. Postoperative immobilization by a splinting cast was only applied for K-wire-treated patients (Table 2). No complications correlated to cast immobilization were reported in the K-wire-treated patients.

In one study three K-wire-treated patients were reported to have fractured a previously non-injured neighboring metacarpal after a new trauma at a former K-wire site during follow-up period [16].

### Risk of bias and level of evidence

None of the selected studies compared two types of treatment. All five studies reported on cohorts of patients treated by one type of fixation. All five studies were therefore graded levels of evidence 4 (Table 1) [17]. Relevant types of potential bias within these studies included selection bias and follow-up bias.

Although all five studies only report on one type of surgical technique a selection bias might be considered. Because all included patients in this review were included for surgery based on the same surgical indication; a single, closed metacarpal shaft fractures with rotational deformity, comparison of the reported results is possible.

Follow-up bias might be considered in the selected studies, as the follow-up period was not reported in one study [13]. As all patients in this study were reported to have a full function outcome this possible bias does not influence comparison of the reported results.

## Discussion

No randomized or non-randomized study comparing ORIF with K-wire fixation was found. Based on the data from the included literature, reporting on a total of 101 patients operated for a single, closed unstable metacarpal shaft fracture, complication rates are more frequently found in the K-wire-treated patients (22 vs. 35 % respectively). However, the reported complications after ORIF are more frequently related to functional impairment and more often require reoperation (15 %), whereas most complications after K-wire fixation involved superficial infection, which could be treated conservatively.

Second, Fusetti et al. [4] suggest that exploration for ORIF results in loss of the fracture hematoma, which may give rise to delayed union and non-union. As no consolidation problems were reported this suggestion cannot be confirmed. On basis of the included data there does not seem to be any evidence for fracture healing problems in the treatment of a single, closed metacarpal shaft fractures with ORIF or with K-wire fixation.

Although the general functional outcome was reported to be good for both techniques, the data shows one ORIF-treated patient with a DASH score of 44 [19]. Such a DASH score is likely to be associated with loss of function. Unfortunately no further specifications are made and no explanation is given for this finding by the authors. Similarly, one K-wire patient also scored a relative high DASH score of 39. The authors suggest a plausible reason by explaining the patient had encountered additional injuries to the upper limb, non related to the operated hand, but therefore possibly resulting in a biased DASH score.

One of the limitations of this systematic review is the lack of comparative studies on outcomes after ORIF and K-wire fixation of single, closed metacarpal shaft fractures of the second to fifth metacarpal. Comparison of outcome between the data from the included articles is appropriate as similar indications for surgery have been applied in all five articles. A second limitation is the lack of patients treated solely with screw fixation. Possibly less dissection is required for this type of fixation in comparison to plate fixation.

In contrast to earlier publications postoperative immobilization did not influence postoperative functional repair in the reported studies. Postoperative cast immobilization was only applied after K-wire fixation and was reported to be associated with good functional results. None of the ORIF-treated patients were immobilized with cats (Table 2). Therefore, cast immobilization cannot have been a reason for the development of functional impairment requiring reoperations as found in these ORIF-treated patients. Also cast immobilization can be safely applied, without increased chance of functional impairment, in K-wire-treated patients for support of soft tissue and fracture healing the first weeks after surgery (Tables 2, 3).

No specification was made about the type of fracture, other than shaft fractures located in the second, third, fourth or fifth metacarpal (Table 2). All fractures were operated because of instability, angulations’ or rotational deformity. No comparison can therefore be made between fracture type (i.e. spiral, oblique) and functional results. As all studies reported identical indication for surgical fixation a comparison between type of fixation and functional result can be made.

Based on the reported results there is no level I evidence to suggest one fixation technique over another. The reported complications however for ORIF and K-wire fixation in the treatment of single, closed metacarpal shaft fractures are unmistakably different for the two types of fixation. ORIF was associated with a considerable number of functional restricting complications and consequent reoperations, whereas K-wire fixation resulted frequently in superficial infection treated conservatively. The significance of these reported findings suggest ORIF might be a less preferable surgical technique in comparison to K-wire fixation in the treatment of a single metacarpal shaft fracture. To confirm this finding further research is warranted and should focus on the comparison between ORIF and K-wire fixation for single, closed metacarpal shaft fractures, preferably in a randomized clinical trial.

## Appendix : Search strategies

*MedLine*: (“Metacarpal Bones”[Mesh] OR “metacarpal”[all fields] OR “metacarpus”[all fields]) AND (“Fractures, Bone”[Mesh] OR fracture[all fields] OR fractures[all fields] OR “Fracture Fixation”[Mesh] OR “Fracture Healing”[Mesh]) AND (“midshaft”[all fields] OR “shaft”[all fields] OR “mid-shaft”[all fields] OR “middle third”[all fields] OR “diaphysis”[all fields] OR “extra-articular”[all fields]) AND (“Surgical Procedures, Operative”[Mesh] OR “surgery”[all fields] OR “surgical”[all fields] OR “operative”[all fields] OR “Orthopedic Fixation Devices”[Mesh] OR “fixation”[all fields] OR “fixator”[all fields] OR “fixators”[all fields]).

*Embase*: (((metacarpal bone/OR “metacarpal”.mp. OR “metacarpus”.mp.) AND (exp fracture/OR fracture.mp. OR fractures.mp. OR exp fracture fixation/OR fracture treatment/OR fracture healing/OR fracture reduction/)) OR metacarpal bone fracture/) AND (“midshaft”.mp. OR “shaft”.mp. OR “mid-shaft”.mp. OR “middle third”.mp. OR “diaphysis”.mp. OR “extra-articular”.mp.) AND (exp surgery/OR “surgery”.mp. OR “surgical”.mp. OR “operative”.mp. OR fixation device/OR exp fracture fixation/OR “fixation”.mp. OR “fixator”.mp. OR “fixators”.mp.).
